# μ-4,4′-Bipyridyl-1:2κ^2^ *N*:*N*′-methanol-2κ*O*-tetrakis(tri-*tert*-butoxy­silanethiol­ato)-1κ^4^ *O*,*S*;2κ^2^ *S*-dizinc(II)

**DOI:** 10.1107/S1600536808008210

**Published:** 2008-04-02

**Authors:** Anna Dołęga, Katarzyna Baranowska

**Affiliations:** a Department of Inorganic Chemistry, Faculty of Chemistry, Gdańsk University of Technology, 11/12 G. Narutowicz Street, 80952-PL Gdańsk, Poland

## Abstract

The title compound, [Zn_2_(C_12_H_27_O_3_SSi)_4_(C_10_H_8_N_2_)(CH_4_O)], is a binuclear complex with the two Zn^II^ atoms linked *via* a bridging 4,4′-bipyridyl ligand. One of the Zn^II^ atoms is penta-coordinated by two *O*,*S*-chelating tri-*tert*-butoxy­silanethiol­ate units and one N atom of a 4,4′-bipyridine ligand, and the other Zn^II^ atom is tetra­hedrally coordinated by two tri-*tert*-butoxy­silanethiol­ate anions acting as monodentate S ligands, the methanol O atom and the other N atom of the 4,4′-bipyridine ligand. This non-symmetrical coordination induces twisting and bending in the 4,4′-bipyridine ligand and introduces chirality into the system. The crystal studied exhibits inversion twinning. One *tert*-butyl group is disordered approximately equally over two positions.

## Related literature

For related literature, see: Bąkowicz *et al.* (2007 ▶); Becker *et al.* (2001 ▶); Pladzyk *et al.* (2007 ▶).
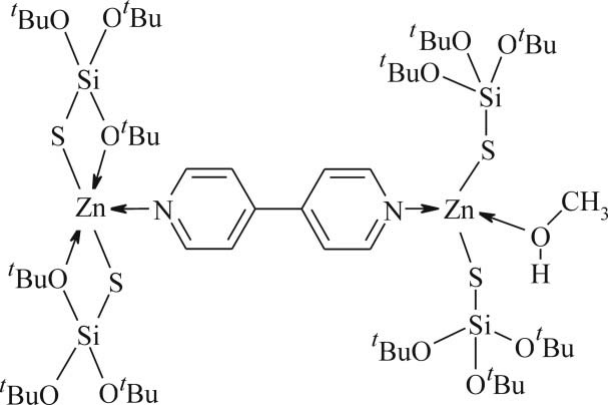


## Experimental

### 

#### Crystal data

[Zn_2_(C_12_H_27_O_3_SSi)_4_(C_10_H_8_N_2_)(CH_4_O)]
*M* *_r_* = 1436.91
Monoclinic, 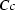

*a* = 9.5897 (2) Å
*b* = 36.8378 (11) Å
*c* = 22.7611 (5) Å
β = 99.127 (2)°
*V* = 7938.9 (3) Å^3^
*Z* = 4
Mo *K*α radiation
μ = 0.82 mm^−1^
*T* = 120 (2) K
0.31 × 0.12 × 0.10 mm

#### Data collection

Oxford Diffraction KM4CCD diffractometer
Absorption correction: none
15025 measured reflections
9143 independent reflections
8015 reflections with *I* > 2σ(*I*)
*R*_int_ = 0.037

#### Refinement

*R*[*F* ^2^ > 2σ(*F* ^2^)] = 0.044
*wR*(*F* ^2^) = 0.126
*S* = 1.19
9143 reflections
793 parameters
3 restraints
H atoms treated by a mixture of independent and constrained refinement
Δρ_max_ = 1.13 e Å^−3^
Δρ_min_ = −0.66 e Å^−3^
Absolute structure: Flack (1983 ▶); 2104 Friedel pairs
Flack parameter: 0.462 (16)

### 

Data collection: *CrysAlis CCD* (Oxford Diffraction, 2006 ▶); cell refinement: *CrysAlis RED* (Oxford Diffraction, 2006 ▶); data reduction: *CrysAlis RED*; program(s) used to solve structure: *SHELXS97* (Sheldrick, 2008 ▶); program(s) used to refine structure: *SHELXL97* (Sheldrick, 2008 ▶); molecular graphics: *ORTEP-3 for Windows* (Farrugia, 1997 ▶); software used to prepare material for publication: *WinGX* (Farrugia, 1999 ▶).

## Supplementary Material

Crystal structure: contains datablocks I, global. DOI: 10.1107/S1600536808008210/ci2570sup1.cif

Structure factors: contains datablocks I. DOI: 10.1107/S1600536808008210/ci2570Isup2.hkl

Additional supplementary materials:  crystallographic information; 3D view; checkCIF report

## Figures and Tables

**Table:** 

Zn1—N1	2.075 (5)
Zn1—S2	2.2664 (18)
Zn1—S1	2.2732 (16)
Zn1—O1	2.357 (4)
Zn1—O4	2.554 (4)
Zn2—N2	2.098 (5)
Zn2—O13	2.156 (5)
Zn2—S4	2.2627 (16)
Zn2—S3	2.2704 (17)

**Table:** 

N1—Zn1—S2	111.39 (14)
N1—Zn1—S1	107.81 (15)
S2—Zn1—S1	139.71 (7)
N1—Zn1—O1	92.46 (17)
S2—Zn1—O1	108.85 (12)
S1—Zn1—O1	77.94 (11)
N1—Zn1—O4	90.91 (17)
S2—Zn1—O4	74.68 (10)
S1—Zn1—O4	96.04 (11)
O1—Zn1—O4	173.77 (14)
N2—Zn2—O13	93.72 (19)
N2—Zn2—S4	105.61 (15)
O13—Zn2—S4	99.79 (13)
N2—Zn2—S3	109.24 (15)
O13—Zn2—S3	100.81 (13)
S4—Zn2—S3	137.87 (6)

**Table 2:** Hydrogen-bond geometry (Å, °)

*D*—H⋯*A*	*D*—H	H⋯*A*	*D*⋯*A*	*D*—H⋯*A*
O13—H13*A*⋯O10	0.99 (1)	1.86 (4)	2.771 (6)	151 (6)

**Fig. 1.**
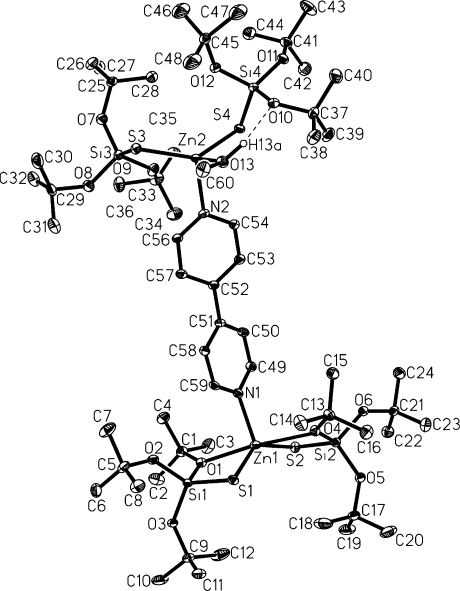
A view of the title molecule, showing the atom-labelling scheme. Displacement ellipsoids are drawn at the 30% probability level. All H atoms except H13A have been omitted for clarity.

## supplementary crystallographic information

### Comment

Two different coordination patterns are observed for zinc atoms in the title binuclear complex. Atom Zn1 is pentacoordinated by two O,*S*–chelating tri-*tert*-butoxysilanethiolate moieties and one N atom of a 4,4'-bipyridine ligand. Atom Zn2 is tetrahedrally coordinated by two tri-*tert*-butoxysilanethiolate anions acting as monodentate S-ligands, methanol oxygen and the other N of the 4,4'-bipyridine ligand (Table 1). This non-symmetric coordination results in a significant distortion of the pyridine rings of the 4,4'-bipyridine ligand; they are not only twisted [torsion angle C50—C51—C52—C53 = -28.8 (10)° and C58—C51—C52—C57 - 30.0 (9)°] but also bent [dihedral angle 31.7 (3)°] as well. The system is chiral and twinned by inversion with the Flack parameter of 0.462 (16) indicating almost equal shares of two crystal domains.

Coordination of methanol on zinc is strengthened by an intramolecular O—H···O hydrogen bonding with one of the adjacent *tert*-butoxy groups (Fig. 1).

### Experimental

The title compound was obtained by the reaction of zinc acetyloacetonate (0.54 g, 2 mmol), 4,4'-bipyridine (0.312 g, 2 mmol) and tri-*tert*-butoxysilanethiol (0.9 ml, 3 mmol) in methanol (30 ml) and recrystallized from methanol at ambient temperature. For synthesis of substrates see, Becker *et al.* (2001).

### Refinement

The *^t^*Bu group attached to O9 is disordered over two orientations with respect to rotation about O9—C33 bond. The site occupancies of the disordered atoms C34,C35,C36/C34A,C35A,C36A were refined to 0.52 (2)/0.48 (2). The same anisotropic displacement parameters were used for atoms C29, C30, C31 and C32, and for atoms C45, C46, C47 and C48. The hydroxyl H atom was located in a difference map and its positional parameters were refined with a O—H distance restraint of 0.99 (1) Å. The remaining H atoms were positioned geometrically and refined using a riding model, with C–H 0.95–0.98 Å and *U*_iso_(H) = 1.2*U*_eq_(C,*O*) or 1.5*U*_eq_(methyl C). The highest residual electron-density peak is located 0.04 Å from atom C45.

### Crystal data

**Table:**

[Zn_2_(C_12_H_27_O_3_SSi)_4_(C_10_H_8_N_2_)(CH_4_O)]	*F*_000_ = 3088
*M**_r_* = 1436.91	*D*_x_ = 1.202 Mg m^−^^3^
Monoclinic, *C**c*	Mo *K*α radiation λ = 0.71073 Å
Hall symbol: C -2yc	Cell parameters from 12077 reflections
*a* = 9.5897 (2) Å	θ = 2.2–32.5º
*b* = 36.8378 (11) Å	µ = 0.82 mm^−^^1^
*c* = 22.7611 (5) Å	*T* = 120 (2) K
β = 99.127 (2)º	Prism, colourless
*V* = 7938.9 (3) Å^3^	0.31 × 0.12 × 0.10 mm
*Z* = 4	

### Data collection

**Table:**

Oxford Diffraction KM-4-CCD diffractometer	8015 reflections with *I* > 2σ(*I*)
Monochromator: graphite	*R*_int_ = 0.037
Detector resolution: 8.1883 pixels mm^-1^	θ_max_ = 25.1º
*T* = 120(2) K	θ_min_ = 2.2º
ω scans, 0.70 deg width	*h* = −7→11
Absorption correction: none	*k* = −33→43
15025 measured reflections	*l* = −27→27
9143 independent reflections	

### Refinement

**Table:**

Refinement on *F*^2^	Hydrogen site location: inferred from neighbouring sites
Least-squares matrix: full	H atoms treated by a mixture of independent and constrained refinement
*R*[*F*^2^ > 2σ(*F*^2^)] = 0.044	*w* = 1/[σ^2^(*F*_o_^2^) + (0.071*P*)^2^ + 7.4679*P*] where *P* = (*F*_o_^2^ + 2*F*_c_^2^)/3
*wR*(*F*^2^) = 0.126	(Δ/σ)_max_ = 0.013
*S* = 1.19	Δρ_max_ = 1.13 e Å^−^^3^
9143 reflections	Δρ_min_ = −0.66 e Å^−^^3^
793 parameters	Extinction correction: none
3 restraints	Absolute structure: Flack (1983); 2104 Friedel pairs
Primary atom site location: structure-invariant direct methods	Flack parameter: 0.462 (16)
Secondary atom site location: difference Fourier map	

### Special details

**Table:**

Geometry. All e.s.d.'s (except the e.s.d. in the dihedral angle between two l.s. planes) are estimated using the full covariance matrix. The cell e.s.d.'s are taken into account individually in the estimation of e.s.d.'s in distances, angles and torsion angles; correlations between e.s.d.'s in cell parameters are only used when they are defined by crystal symmetry. An approximate (isotropic) treatment of cell e.s.d.'s is used for estimating e.s.d.'s involving l.s. planes.
Refinement. Refinement of *F*^2^ against ALL reflections. The weighted *R*-factor *wR* and goodness of fit *S* are based on *F*^2^, conventional *R*-factors *R* are based on *F*, with *F* set to zero for negative *F*^2^. The threshold expression of *F*^2^ > σ(*F*^2^) is used only for calculating *R*-factors(gt) *etc*. and is not relevant to the choice of reflections for refinement. *R*-factors based on *F*^2^ are statistically about twice as large as those based on *F*, and *R*- factors based on ALL data will be even larger.

### Fractional atomic coordinates and isotropic or equivalent isotropic displacement parameters (Å^2^)

**Table:**

	*x*	*y*	*z*	*U*_iso_*/*U*_eq_	Occ. (<1)
Zn1	0.87328 (8)	0.419112 (17)	0.52174 (3)	0.02354 (17)	
Zn2	0.02975 (8)	0.208801 (17)	0.49213 (3)	0.02269 (17)	
S1	0.75131 (17)	0.47221 (4)	0.50965 (7)	0.0238 (3)	
S2	1.08592 (17)	0.39609 (4)	0.50945 (7)	0.0243 (3)	
S3	−0.13485 (16)	0.22289 (4)	0.54999 (7)	0.0242 (3)	
S4	0.12611 (17)	0.15828 (4)	0.45870 (7)	0.0255 (3)	
Si1	0.77984 (18)	0.47450 (4)	0.60187 (7)	0.0198 (3)	
Si2	1.02088 (18)	0.39671 (4)	0.41728 (7)	0.0214 (4)	
Si3	−0.00917 (18)	0.20566 (4)	0.62862 (8)	0.0222 (4)	
Si4	−0.04263 (19)	0.14141 (4)	0.39600 (8)	0.0226 (4)	
O1	0.8867 (5)	0.43946 (11)	0.62064 (18)	0.0235 (9)	
O2	0.6414 (5)	0.46546 (11)	0.6328 (2)	0.0259 (10)	
O3	0.8409 (5)	0.51256 (11)	0.63097 (18)	0.0260 (10)	
O4	0.8502 (4)	0.40405 (11)	0.41111 (18)	0.0244 (9)	
O5	1.1011 (5)	0.42614 (11)	0.38143 (19)	0.0268 (10)	
O6	1.0387 (5)	0.35829 (11)	0.3837 (2)	0.0262 (10)	
O7	−0.0682 (5)	0.17051 (12)	0.6606 (2)	0.0304 (10)	
O8	0.0160 (5)	0.23723 (12)	0.6794 (2)	0.0336 (11)	
O9	0.1467 (5)	0.19839 (12)	0.6113 (2)	0.0327 (11)	
O10	−0.0793 (5)	0.17008 (11)	0.3394 (2)	0.0288 (10)	
O11	−0.0115 (5)	0.10291 (10)	0.36563 (19)	0.0290 (10)	
O12	−0.1833 (5)	0.14153 (12)	0.42733 (19)	0.0291 (10)	
N1	0.7317 (5)	0.37820 (12)	0.5337 (2)	0.0212 (11)	
N2	0.1936 (6)	0.24711 (13)	0.5063 (2)	0.0246 (12)	
C1	0.9323 (8)	0.42254 (17)	0.6785 (3)	0.0296 (15)	
C2	0.9660 (11)	0.4513 (2)	0.7258 (4)	0.052 (2)	
H2A	1.0365	0.4681	0.7144	0.078*	
H2B	1.0036	0.4398	0.7639	0.078*	
H2C	0.8798	0.4648	0.7299	0.078*	
C3	1.0618 (8)	0.4007 (2)	0.6716 (4)	0.0427 (19)	
H3A	1.0358	0.3814	0.6422	0.064*	
H3B	1.1015	0.3898	0.7099	0.064*	
H3C	1.1323	0.4166	0.658	0.064*	
C4	0.8154 (9)	0.39783 (19)	0.6933 (3)	0.0400 (17)	
H4A	0.732	0.4124	0.6974	0.06*	
H4B	0.8481	0.3851	0.7308	0.06*	
H4C	0.7908	0.3801	0.6613	0.06*	
C5	0.5252 (8)	0.48737 (18)	0.6439 (3)	0.0345 (16)	
C6	0.5703 (10)	0.5089 (2)	0.7006 (4)	0.048 (2)	
H6A	0.6128	0.4925	0.7324	0.072*	
H6B	0.4877	0.5208	0.7125	0.072*	
H6C	0.6396	0.5273	0.6937	0.072*	
C7	0.4094 (9)	0.4608 (2)	0.6530 (5)	0.053 (2)	
H7A	0.3749	0.4484	0.6154	0.08*	
H7B	0.3315	0.4739	0.6664	0.08*	
H7C	0.4473	0.4428	0.6831	0.08*	
C8	0.4762 (8)	0.5125 (2)	0.5918 (4)	0.0421 (18)	
H8A	0.5515	0.5298	0.5875	0.063*	
H8B	0.392	0.5257	0.5991	0.063*	
H8C	0.4536	0.4982	0.5553	0.063*	
C9	0.9372 (7)	0.53963 (17)	0.6156 (3)	0.0288 (15)	
C10	0.9754 (12)	0.5622 (2)	0.6713 (4)	0.058 (3)	
H10A	0.8898	0.5733	0.6818	0.086*	
H10B	1.0419	0.5813	0.6641	0.086*	
H10C	1.0192	0.5466	0.704	0.086*	
C11	0.8618 (9)	0.56240 (18)	0.5651 (3)	0.0369 (17)	
H11A	0.8297	0.5468	0.5308	0.055*	
H11B	0.9267	0.5808	0.5541	0.055*	
H11C	0.7803	0.5744	0.5778	0.055*	
C12	1.0658 (9)	0.5221 (2)	0.5975 (5)	0.062 (3)	
H12A	1.1144	0.5075	0.6306	0.092*	
H12B	1.1298	0.541	0.5871	0.092*	
H12C	1.0369	0.5064	0.5629	0.092*	
C13	0.7380 (7)	0.39982 (17)	0.3603 (3)	0.0274 (14)	
C14	0.6177 (8)	0.4239 (2)	0.3728 (4)	0.0407 (18)	
H14A	0.5851	0.4158	0.4093	0.061*	
H14B	0.5396	0.4225	0.3394	0.061*	
H14C	0.6507	0.4491	0.3777	0.061*	
C15	0.6936 (8)	0.35970 (18)	0.3574 (3)	0.0345 (16)	
H15A	0.7749	0.3445	0.3524	0.052*	
H15B	0.618	0.356	0.3236	0.052*	
H15C	0.6596	0.353	0.3943	0.052*	
C16	0.7898 (8)	0.4118 (2)	0.3033 (3)	0.0407 (18)	
H16A	0.8273	0.4366	0.3083	0.061*	
H16B	0.7109	0.4113	0.2702	0.061*	
H16C	0.8643	0.3954	0.2949	0.061*	
C17	1.1433 (8)	0.46377 (18)	0.3905 (3)	0.0329 (16)	
C18	1.0280 (10)	0.4857 (2)	0.4107 (5)	0.062 (3)	
H18A	0.9399	0.4822	0.3829	0.093*	
H18B	1.0538	0.5115	0.4117	0.093*	
H18C	1.0149	0.4779	0.4506	0.093*	
C19	1.2794 (10)	0.4656 (2)	0.4338 (4)	0.053 (2)	
H19A	1.2637	0.4565	0.4727	0.08*	
H19B	1.3119	0.4908	0.4377	0.08*	
H19C	1.3511	0.4507	0.4191	0.08*	
C20	1.1674 (12)	0.4766 (2)	0.3301 (4)	0.061 (3)	
H20A	1.2357	0.4606	0.3152	0.091*	
H20B	1.2043	0.5014	0.3331	0.091*	
H20C	1.0779	0.4761	0.3025	0.091*	
C21	1.1553 (7)	0.34331 (16)	0.3591 (3)	0.0277 (14)	
C22	1.2936 (8)	0.3511 (2)	0.3992 (4)	0.0384 (17)	
H22A	1.312	0.3772	0.3999	0.058*	
H22B	1.3703	0.3383	0.384	0.058*	
H22C	1.2881	0.3426	0.4396	0.058*	
C23	1.1565 (9)	0.3581 (2)	0.2971 (3)	0.0420 (18)	
H23A	1.071	0.3502	0.2708	0.063*	
H23B	1.24	0.349	0.2819	0.063*	
H23C	1.1594	0.3847	0.2985	0.063*	
C24	1.1282 (9)	0.30224 (18)	0.3556 (4)	0.0389 (17)	
H24A	1.1268	0.2927	0.3957	0.058*	
H24B	1.2034	0.2903	0.3383	0.058*	
H24C	1.0369	0.2975	0.3307	0.058*	
C25	−0.1345 (7)	0.13685 (15)	0.6370 (3)	0.0261 (14)	
C26	−0.0969 (9)	0.10901 (19)	0.6873 (3)	0.0394 (18)	
H26A	0.0055	0.1051	0.6944	0.059*	
H26B	−0.145	0.086	0.6758	0.059*	
H26C	−0.1272	0.1182	0.7237	0.059*	
C27	−0.2920 (8)	0.14394 (19)	0.6247 (3)	0.0350 (16)	
H27A	−0.3245	0.1516	0.6614	0.053*	
H27B	−0.3415	0.1217	0.6099	0.053*	
H27C	−0.3121	0.1631	0.5946	0.053*	
C28	−0.0792 (8)	0.12438 (18)	0.5822 (3)	0.0333 (16)	
H28A	−0.1017	0.1425	0.5506	0.05*	
H28B	−0.1232	0.1012	0.5688	0.05*	
H28C	0.0235	0.1213	0.5913	0.05*	
C29	−0.0737 (11)	0.2549 (3)	0.7148 (5)	0.0671 (14)	
C30	−0.2263 (10)	0.2463 (3)	0.6977 (5)	0.0671 (14)	
H30A	−0.2587	0.2547	0.6569	0.101*	
H30B	−0.2803	0.2587	0.7249	0.101*	
H30C	−0.2404	0.2201	0.6999	0.101*	
C31	−0.0402 (11)	0.2948 (3)	0.7126 (5)	0.0671 (14)	
H31A	0.0621	0.2984	0.7227	0.101*	
H31B	−0.0878	0.3079	0.7413	0.101*	
H31C	−0.0731	0.3042	0.6725	0.101*	
C32	−0.0275 (11)	0.2409 (3)	0.7777 (5)	0.0671 (14)	
H32A	−0.0693	0.217	0.7818	0.101*	
H32B	−0.0593	0.2578	0.8061	0.101*	
H32C	0.0757	0.239	0.7856	0.101*	
C33	0.2783 (7)	0.1869 (2)	0.6471 (4)	0.0407 (18)	
C34	0.399 (3)	0.2111 (7)	0.6362 (11)	0.046 (5)	0.52 (2)
H34A	0.4824	0.206	0.6661	0.07*	0.52 (2)
H34B	0.3719	0.2366	0.6391	0.07*	0.52 (2)
H34C	0.4218	0.2064	0.5964	0.07*	0.52 (2)
C35	0.3074 (16)	0.1498 (4)	0.6381 (8)	0.036 (4)	0.52 (2)
H35A	0.3988	0.1434	0.6615	0.054*	0.52 (2)
H35B	0.3104	0.1456	0.5958	0.054*	0.52 (2)
H35C	0.2332	0.1348	0.6507	0.054*	0.52 (2)
C36	0.2727 (17)	0.1917 (5)	0.7189 (7)	0.044 (5)	0.52 (2)
H36A	0.27	0.2175	0.7287	0.066*	0.52 (2)
H36B	0.3568	0.1806	0.7421	0.066*	0.52 (2)
H36C	0.1879	0.1797	0.7286	0.066*	0.52 (2)
C34A	0.365 (3)	0.2192 (6)	0.6588 (12)	0.045 (6)	0.48 (2)
H34D	0.4557	0.2127	0.6825	0.067*	0.48 (2)
H34E	0.3162	0.2368	0.6808	0.067*	0.48 (2)
H34F	0.3805	0.2299	0.621	0.067*	0.48 (2)
C35A	0.3435 (18)	0.1624 (5)	0.5985 (9)	0.044 (6)	0.48 (2)
H35D	0.4311	0.1508	0.6177	0.065*	0.48 (2)
H35E	0.3636	0.178	0.5659	0.065*	0.48 (2)
H35F	0.2751	0.1437	0.5827	0.065*	0.48 (2)
C36A	0.2532 (19)	0.1649 (6)	0.6954 (10)	0.058 (7)	0.48 (2)
H36D	0.1923	0.178	0.719	0.088*	0.48 (2)
H36E	0.3433	0.1592	0.7206	0.088*	0.48 (2)
H36F	0.2067	0.1423	0.6802	0.088*	0.48 (2)
C37	−0.0162 (8)	0.17337 (18)	0.2852 (3)	0.0321 (16)	
C38	−0.0609 (9)	0.20988 (19)	0.2604 (4)	0.0411 (18)	
H38A	−0.1642	0.2111	0.2521	0.062*	
H38B	−0.0226	0.2138	0.2234	0.062*	
H38C	−0.025	0.2287	0.2893	0.062*	
C39	0.1417 (9)	0.1706 (2)	0.2994 (4)	0.0427 (19)	
H39A	0.1778	0.1894	0.3283	0.064*	
H39B	0.1823	0.174	0.2629	0.064*	
H39C	0.168	0.1466	0.3161	0.064*	
C40	−0.0791 (11)	0.1440 (2)	0.2413 (3)	0.051 (2)	
H40A	−0.0577	0.12	0.2591	0.077*	
H40B	−0.0383	0.1461	0.2046	0.077*	
H40C	−0.1817	0.1472	0.2322	0.077*	
C41	0.0531 (8)	0.06921 (17)	0.3891 (3)	0.0327 (16)	
C42	0.2117 (9)	0.0722 (2)	0.3932 (4)	0.046 (2)	
H42A	0.235	0.0791	0.3543	0.069*	
H42B	0.2554	0.0488	0.4054	0.069*	
H42C	0.2475	0.0908	0.4227	0.069*	
C43	−0.0073 (12)	0.04046 (19)	0.3439 (4)	0.058 (2)	
H43A	−0.1103	0.0397	0.3413	0.087*	
H43B	0.0325	0.0167	0.3566	0.087*	
H43C	0.0169	0.0465	0.3048	0.087*	
C44	0.0133 (9)	0.06086 (18)	0.4498 (3)	0.0386 (18)	
H44A	0.051	0.0798	0.4781	0.058*	
H44B	0.053	0.0373	0.4638	0.058*	
H44C	−0.0898	0.0601	0.4466	0.058*	
C45	−0.3318 (11)	0.1365 (3)	0.4069 (5)	0.0726 (15)	
C46	−0.3974 (10)	0.1729 (3)	0.4057 (5)	0.0726 (15)	
H46A	−0.3683	0.185	0.4441	0.109*	
H46B	−0.5005	0.1705	0.3982	0.109*	
H46C	−0.3669	0.1875	0.374	0.109*	
C47	−0.3877 (10)	0.1155 (3)	0.4557 (5)	0.0726 (15)	
H47A	−0.3379	0.0923	0.4621	0.109*	
H47B	−0.489	0.111	0.4438	0.109*	
H47C	−0.3724	0.1297	0.4926	0.109*	
C48	−0.3621 (10)	0.1192 (3)	0.3481 (5)	0.0726 (15)	
H48A	−0.3231	0.1341	0.3189	0.109*	
H48B	−0.4645	0.117	0.3362	0.109*	
H48C	−0.3189	0.0951	0.3499	0.109*	
C49	0.7655 (7)	0.34292 (15)	0.5319 (3)	0.0252 (14)	
H49	0.8607	0.3366	0.53	0.03*	
C50	0.6686 (7)	0.31558 (16)	0.5325 (3)	0.0256 (14)	
H50	0.6969	0.2909	0.5313	0.031*	
C51	0.5279 (7)	0.32437 (15)	0.5350 (3)	0.0232 (13)	
C52	0.4154 (7)	0.29640 (16)	0.5279 (3)	0.0251 (14)	
C53	0.4244 (7)	0.26550 (17)	0.4945 (3)	0.0294 (15)	
H53	0.5077	0.2607	0.4782	0.035*	
C54	0.3124 (7)	0.24173 (17)	0.4849 (3)	0.0272 (15)	
H54	0.3208	0.2205	0.4619	0.033*	
C56	0.1850 (7)	0.27639 (16)	0.5404 (3)	0.0272 (14)	
H56	0.1014	0.28	0.5572	0.033*	
C57	0.2920 (7)	0.30154 (16)	0.5523 (3)	0.0286 (15)	
H57	0.2821	0.3221	0.5765	0.034*	
C58	0.4954 (7)	0.36096 (15)	0.5397 (3)	0.0222 (13)	
H58	0.4023	0.3681	0.5438	0.027*	
C59	0.5983 (7)	0.38676 (16)	0.5385 (3)	0.0265 (14)	
H59	0.5739	0.4116	0.541	0.032*	
C60	−0.1725 (9)	0.2591 (2)	0.4021 (4)	0.0415 (18)	
H60A	−0.2562	0.2493	0.4163	0.062*	
H60B	−0.1969	0.2658	0.3601	0.062*	
H60C	−0.1388	0.2806	0.4255	0.062*	
O13	−0.0646 (5)	0.23231 (11)	0.4085 (2)	0.0299 (10)	
H13A	−0.076 (8)	0.2162 (16)	0.3734 (19)	0.036*	

### Atomic displacement parameters (Å^2^)

**Table:**

	*U*^11^	*U*^22^	*U*^33^	*U*^12^	*U*^13^	*U*^23^
Zn1	0.0201 (4)	0.0164 (3)	0.0347 (4)	−0.0013 (3)	0.0061 (3)	0.0003 (3)
Zn2	0.0219 (4)	0.0166 (3)	0.0305 (4)	−0.0032 (3)	0.0070 (3)	−0.0025 (3)
S1	0.0293 (8)	0.0179 (7)	0.0232 (8)	0.0019 (6)	0.0014 (7)	0.0007 (6)
S2	0.0197 (7)	0.0307 (8)	0.0220 (8)	0.0022 (7)	0.0015 (6)	−0.0003 (6)
S3	0.0215 (8)	0.0227 (7)	0.0289 (8)	0.0030 (6)	0.0053 (6)	0.0010 (6)
S4	0.0224 (8)	0.0201 (7)	0.0344 (9)	−0.0028 (6)	0.0052 (7)	−0.0070 (6)
Si1	0.0220 (8)	0.0161 (7)	0.0213 (8)	−0.0018 (7)	0.0037 (7)	0.0007 (6)
Si2	0.0199 (8)	0.0228 (8)	0.0213 (9)	−0.0025 (7)	0.0024 (7)	0.0000 (6)
Si3	0.0190 (8)	0.0197 (8)	0.0286 (9)	0.0022 (7)	0.0057 (7)	−0.0007 (7)
Si4	0.0254 (9)	0.0191 (8)	0.0242 (9)	−0.0060 (7)	0.0068 (7)	−0.0028 (7)
O1	0.025 (2)	0.023 (2)	0.020 (2)	−0.0015 (18)	−0.0018 (18)	0.0022 (17)
O2	0.028 (2)	0.018 (2)	0.032 (2)	−0.0014 (18)	0.008 (2)	0.0051 (17)
O3	0.032 (3)	0.021 (2)	0.026 (2)	−0.0078 (19)	0.007 (2)	−0.0009 (17)
O4	0.021 (2)	0.030 (2)	0.022 (2)	0.0007 (19)	0.0027 (18)	−0.0019 (18)
O5	0.031 (2)	0.024 (2)	0.025 (2)	−0.0058 (19)	0.003 (2)	0.0005 (18)
O6	0.023 (2)	0.025 (2)	0.031 (2)	−0.0030 (19)	0.0070 (19)	−0.0006 (18)
O7	0.034 (3)	0.029 (2)	0.029 (2)	−0.004 (2)	0.006 (2)	0.0001 (19)
O8	0.026 (2)	0.036 (3)	0.038 (3)	0.000 (2)	0.005 (2)	−0.010 (2)
O9	0.024 (2)	0.037 (3)	0.039 (3)	0.007 (2)	0.009 (2)	0.008 (2)
O10	0.034 (3)	0.022 (2)	0.031 (2)	−0.004 (2)	0.007 (2)	−0.0002 (18)
O11	0.042 (3)	0.021 (2)	0.024 (2)	−0.005 (2)	0.004 (2)	−0.0030 (17)
O12	0.027 (2)	0.035 (2)	0.027 (2)	−0.009 (2)	0.008 (2)	−0.0058 (19)
N1	0.019 (3)	0.017 (2)	0.027 (3)	−0.001 (2)	0.004 (2)	−0.004 (2)
N2	0.025 (3)	0.021 (3)	0.028 (3)	−0.006 (2)	0.005 (2)	0.003 (2)
C1	0.033 (4)	0.032 (3)	0.022 (3)	0.000 (3)	0.000 (3)	0.005 (3)
C2	0.078 (6)	0.039 (4)	0.032 (4)	−0.003 (4)	−0.013 (4)	0.004 (3)
C3	0.039 (4)	0.049 (4)	0.041 (4)	0.017 (4)	0.006 (4)	0.022 (3)
C4	0.047 (5)	0.037 (4)	0.037 (4)	−0.002 (4)	0.008 (4)	0.013 (3)
C5	0.032 (4)	0.028 (3)	0.048 (4)	0.003 (3)	0.020 (3)	0.010 (3)
C6	0.070 (6)	0.037 (4)	0.045 (5)	0.007 (4)	0.031 (4)	0.004 (3)
C7	0.031 (4)	0.049 (5)	0.085 (7)	0.000 (4)	0.025 (4)	0.024 (4)
C8	0.029 (4)	0.040 (4)	0.059 (5)	0.009 (3)	0.012 (4)	0.010 (4)
C9	0.033 (4)	0.024 (3)	0.028 (4)	−0.011 (3)	0.000 (3)	0.003 (3)
C10	0.088 (7)	0.048 (5)	0.034 (4)	−0.043 (5)	0.003 (4)	−0.003 (4)
C11	0.043 (4)	0.026 (4)	0.041 (4)	−0.005 (3)	0.003 (3)	0.002 (3)
C12	0.029 (4)	0.047 (5)	0.109 (8)	−0.009 (4)	0.013 (5)	0.020 (5)
C13	0.023 (3)	0.028 (3)	0.028 (4)	−0.002 (3)	−0.005 (3)	−0.003 (3)
C14	0.023 (4)	0.054 (5)	0.042 (4)	0.010 (3)	−0.004 (3)	−0.011 (4)
C15	0.034 (4)	0.042 (4)	0.026 (4)	−0.007 (3)	0.000 (3)	−0.007 (3)
C16	0.036 (4)	0.058 (5)	0.027 (4)	−0.010 (4)	−0.001 (3)	0.002 (3)
C17	0.034 (4)	0.028 (3)	0.036 (4)	−0.011 (3)	0.007 (3)	0.002 (3)
C18	0.057 (6)	0.023 (4)	0.110 (8)	−0.007 (4)	0.024 (6)	−0.003 (4)
C19	0.053 (5)	0.052 (5)	0.050 (5)	−0.028 (4)	−0.003 (4)	−0.001 (4)
C20	0.082 (7)	0.056 (5)	0.042 (5)	−0.035 (5)	0.005 (5)	0.011 (4)
C21	0.027 (3)	0.025 (3)	0.034 (4)	−0.002 (3)	0.014 (3)	0.001 (3)
C22	0.032 (4)	0.036 (4)	0.047 (4)	0.001 (3)	0.005 (3)	−0.003 (3)
C23	0.047 (5)	0.049 (4)	0.032 (4)	0.001 (4)	0.013 (4)	−0.002 (3)
C24	0.040 (4)	0.031 (4)	0.049 (5)	0.004 (3)	0.017 (4)	−0.005 (3)
C25	0.030 (4)	0.017 (3)	0.033 (4)	−0.004 (3)	0.011 (3)	0.007 (2)
C26	0.051 (5)	0.033 (4)	0.037 (4)	0.005 (3)	0.014 (4)	0.008 (3)
C27	0.031 (4)	0.032 (4)	0.044 (4)	0.001 (3)	0.012 (3)	0.008 (3)
C28	0.036 (4)	0.029 (4)	0.036 (4)	0.000 (3)	0.009 (3)	0.000 (3)
C29	0.052 (3)	0.074 (3)	0.081 (3)	0.000 (3)	0.026 (3)	−0.025 (3)
C30	0.052 (3)	0.074 (3)	0.081 (3)	0.000 (3)	0.026 (3)	−0.025 (3)
C31	0.052 (3)	0.074 (3)	0.081 (3)	0.000 (3)	0.026 (3)	−0.025 (3)
C32	0.052 (3)	0.074 (3)	0.081 (3)	0.000 (3)	0.026 (3)	−0.025 (3)
C33	0.017 (3)	0.041 (4)	0.063 (5)	0.008 (3)	0.002 (3)	0.004 (4)
C34	0.036 (12)	0.041 (12)	0.059 (16)	−0.009 (9)	0.000 (10)	0.004 (10)
C35	0.034 (8)	0.026 (7)	0.045 (11)	0.007 (6)	−0.005 (7)	−0.003 (6)
C36	0.033 (8)	0.057 (11)	0.038 (9)	0.010 (8)	−0.003 (7)	−0.005 (8)
C34A	0.044 (15)	0.036 (12)	0.046 (15)	−0.004 (10)	−0.019 (11)	0.000 (10)
C35A	0.029 (9)	0.040 (10)	0.058 (13)	0.018 (7)	−0.003 (8)	−0.021 (9)
C36A	0.028 (9)	0.072 (16)	0.069 (15)	0.001 (9)	−0.012 (9)	0.033 (13)
C37	0.039 (4)	0.032 (4)	0.028 (4)	−0.004 (3)	0.014 (3)	0.004 (3)
C38	0.043 (5)	0.041 (4)	0.041 (4)	0.002 (4)	0.013 (4)	0.009 (3)
C39	0.049 (5)	0.035 (4)	0.050 (5)	0.001 (4)	0.026 (4)	0.014 (3)
C40	0.087 (7)	0.044 (4)	0.027 (4)	−0.018 (4)	0.021 (4)	−0.005 (3)
C41	0.046 (4)	0.021 (3)	0.031 (4)	−0.001 (3)	0.009 (3)	−0.004 (3)
C42	0.051 (5)	0.031 (4)	0.059 (5)	0.005 (4)	0.021 (4)	−0.002 (3)
C43	0.092 (7)	0.026 (4)	0.052 (5)	0.000 (4)	0.002 (5)	−0.015 (4)
C44	0.049 (5)	0.025 (3)	0.043 (4)	0.003 (3)	0.011 (4)	0.008 (3)
C45	0.040 (3)	0.106 (4)	0.068 (3)	−0.020 (3)	−0.002 (2)	−0.009 (3)
C46	0.040 (3)	0.106 (4)	0.068 (3)	−0.020 (3)	−0.002 (2)	−0.009 (3)
C47	0.040 (3)	0.106 (4)	0.068 (3)	−0.020 (3)	−0.002 (2)	−0.009 (3)
C48	0.040 (3)	0.106 (4)	0.068 (3)	−0.020 (3)	−0.002 (2)	−0.009 (3)
C49	0.024 (3)	0.022 (3)	0.028 (3)	0.001 (3)	0.001 (3)	−0.001 (2)
C50	0.022 (3)	0.017 (3)	0.036 (4)	0.003 (3)	0.001 (3)	−0.001 (3)
C51	0.022 (3)	0.019 (3)	0.028 (3)	−0.007 (3)	0.001 (3)	0.000 (2)
C52	0.025 (3)	0.019 (3)	0.031 (4)	−0.006 (3)	0.006 (3)	0.004 (2)
C53	0.023 (3)	0.028 (3)	0.038 (4)	−0.002 (3)	0.008 (3)	−0.005 (3)
C54	0.030 (4)	0.021 (3)	0.033 (4)	−0.004 (3)	0.011 (3)	−0.004 (3)
C56	0.028 (4)	0.021 (3)	0.035 (4)	−0.002 (3)	0.012 (3)	0.001 (3)
C57	0.032 (4)	0.016 (3)	0.039 (4)	−0.001 (3)	0.009 (3)	−0.001 (3)
C58	0.019 (3)	0.020 (3)	0.029 (3)	−0.001 (3)	0.006 (3)	−0.003 (2)
C59	0.027 (4)	0.017 (3)	0.036 (4)	0.002 (3)	0.006 (3)	−0.009 (3)
C60	0.044 (4)	0.039 (4)	0.043 (4)	0.011 (4)	0.011 (4)	0.009 (3)
O13	0.036 (3)	0.024 (2)	0.030 (3)	0.001 (2)	0.007 (2)	0.0027 (18)

### Geometric parameters (Å, °)

**Table:**

Zn1—N1	2.075 (5)	C24—H24A	0.98
Zn1—S2	2.2664 (18)	C24—H24B	0.98
Zn1—S1	2.2732 (16)	C24—H24C	0.98
Zn1—O1	2.357 (4)	C25—C28	1.504 (9)
Zn1—O4	2.554 (4)	C25—C27	1.515 (10)
Zn2—N2	2.098 (5)	C25—C26	1.536 (9)
Zn2—O13	2.156 (5)	C26—H26A	0.98
Zn2—S4	2.2627 (16)	C26—H26B	0.98
Zn2—S3	2.2704 (17)	C26—H26C	0.98
S1—Si1	2.075 (2)	C27—H27A	0.98
S2—Si2	2.092 (2)	C27—H27B	0.98
S3—Si3	2.091 (2)	C27—H27C	0.98
S4—Si4	2.076 (2)	C28—H28A	0.98
Si1—O3	1.620 (4)	C28—H28B	0.98
Si1—O2	1.633 (5)	C28—H28C	0.98
Si1—O1	1.661 (4)	C29—C30	1.487 (14)
Si2—O5	1.622 (5)	C29—C31	1.510 (14)
Si2—O6	1.630 (4)	C29—C32	1.520 (16)
Si2—O4	1.643 (5)	C30—H30A	0.98
Si3—O9	1.628 (5)	C30—H30B	0.98
Si3—O8	1.630 (5)	C30—H30C	0.98
Si3—O7	1.631 (5)	C31—H31A	0.98
Si4—O12	1.623 (5)	C31—H31B	0.98
Si4—O11	1.626 (4)	C31—H31C	0.98
Si4—O10	1.660 (5)	C32—H32A	0.98
O1—C1	1.460 (7)	C32—H32B	0.98
O2—C5	1.430 (8)	C32—H32C	0.98
O3—C9	1.439 (7)	C33—C35	1.417 (15)
O4—C13	1.457 (8)	C33—C36A	1.42 (2)
O5—C17	1.450 (8)	C33—C34A	1.45 (3)
O6—C21	1.438 (8)	C33—C34	1.51 (2)
O7—C25	1.456 (8)	C33—C35A	1.627 (18)
O8—C29	1.425 (10)	C33—C36	1.654 (18)
O9—C33	1.453 (9)	C34—H34A	0.98
O10—C37	1.462 (8)	C34—H34B	0.98
O11—C41	1.451 (8)	C34—H34C	0.98
O12—C45	1.438 (11)	C35—H35A	0.98
N1—C59	1.338 (8)	C35—H35B	0.98
N1—C49	1.342 (8)	C35—H35C	0.98
N2—C54	1.323 (9)	C36—H36A	0.98
N2—C56	1.339 (8)	C36—H36B	0.98
C1—C2	1.510 (10)	C36—H36C	0.98
C1—C3	1.510 (10)	C34A—H34D	0.98
C1—C4	1.523 (10)	C34A—H34E	0.98
C2—H2A	0.98	C34A—H34F	0.98
C2—H2B	0.98	C35A—H35D	0.98
C2—H2C	0.98	C35A—H35E	0.98
C3—H3A	0.98	C35A—H35F	0.98
C3—H3B	0.98	C36A—H36D	0.98
C3—H3C	0.98	C36A—H36E	0.98
C4—H4A	0.98	C36A—H36F	0.98
C4—H4B	0.98	C37—C38	1.495 (10)
C4—H4C	0.98	C37—C39	1.501 (11)
C5—C8	1.518 (10)	C37—C40	1.529 (10)
C5—C6	1.519 (11)	C38—H38A	0.98
C5—C7	1.520 (10)	C38—H38B	0.98
C6—H6A	0.98	C38—H38C	0.98
C6—H6B	0.98	C39—H39A	0.98
C6—H6C	0.98	C39—H39B	0.98
C7—H7A	0.98	C39—H39C	0.98
C7—H7B	0.98	C40—H40A	0.98
C7—H7C	0.98	C40—H40B	0.98
C8—H8A	0.98	C40—H40C	0.98
C8—H8B	0.98	C41—C42	1.513 (11)
C8—H8C	0.98	C41—C44	1.523 (10)
C9—C12	1.507 (11)	C41—C43	1.525 (10)
C9—C10	1.510 (10)	C42—H42A	0.98
C9—C11	1.511 (10)	C42—H42B	0.98
C10—H10A	0.98	C42—H42C	0.98
C10—H10B	0.98	C43—H43A	0.98
C10—H10C	0.98	C43—H43B	0.98
C11—H11A	0.98	C43—H43C	0.98
C11—H11B	0.98	C44—H44A	0.98
C11—H11C	0.98	C44—H44B	0.98
C12—H12A	0.98	C44—H44C	0.98
C12—H12B	0.98	C45—C48	1.469 (14)
C12—H12C	0.98	C45—C46	1.482 (15)
C13—C14	1.518 (9)	C45—C47	1.519 (15)
C13—C16	1.527 (10)	C46—H46A	0.98
C13—C15	1.537 (9)	C46—H46B	0.98
C14—H14A	0.98	C46—H46C	0.98
C14—H14B	0.98	C47—H47A	0.98
C14—H14C	0.98	C47—H47B	0.98
C15—H15A	0.98	C47—H47C	0.98
C15—H15B	0.98	C48—H48A	0.98
C15—H15C	0.98	C48—H48B	0.98
C16—H16A	0.98	C48—H48C	0.98
C16—H16B	0.98	C49—C50	1.372 (9)
C16—H16C	0.98	C49—H49	0.95
C17—C18	1.499 (11)	C50—C51	1.398 (9)
C17—C19	1.506 (11)	C50—H50	0.95
C17—C20	1.508 (10)	C51—C58	1.392 (8)
C18—H18A	0.98	C51—C52	1.482 (8)
C18—H18B	0.98	C52—C53	1.380 (9)
C18—H18C	0.98	C52—C57	1.396 (9)
C19—H19A	0.98	C53—C54	1.376 (9)
C19—H19B	0.98	C53—H53	0.95
C19—H19C	0.98	C54—H54	0.95
C20—H20A	0.98	C56—C57	1.378 (9)
C20—H20B	0.98	C56—H56	0.95
C20—H20C	0.98	C57—H57	0.95
C21—C22	1.513 (10)	C58—C59	1.373 (9)
C21—C23	1.513 (10)	C58—H58	0.95
C21—C24	1.535 (9)	C59—H59	0.95
C22—H22A	0.98	C60—O13	1.419 (9)
C22—H22B	0.98	C60—H60A	0.98
C22—H22C	0.98	C60—H60B	0.98
C23—H23A	0.98	C60—H60C	0.98
C23—H23B	0.98	O13—H13A	0.988 (10)
C23—H23C	0.98		
			
N1—Zn1—S2	111.39 (14)	H24A—C24—H24B	109.5
N1—Zn1—S1	107.81 (15)	C21—C24—H24C	109.5
S2—Zn1—S1	139.71 (7)	H24A—C24—H24C	109.5
N1—Zn1—O1	92.46 (17)	H24B—C24—H24C	109.5
S2—Zn1—O1	108.85 (12)	O7—C25—C28	112.0 (5)
S1—Zn1—O1	77.94 (11)	O7—C25—C27	106.6 (5)
N1—Zn1—O4	90.91 (17)	C28—C25—C27	111.8 (6)
S2—Zn1—O4	74.68 (10)	O7—C25—C26	104.9 (5)
S1—Zn1—O4	96.04 (11)	C28—C25—C26	110.0 (5)
O1—Zn1—O4	173.77 (14)	C27—C25—C26	111.3 (6)
N2—Zn2—O13	93.72 (19)	C25—C26—H26A	109.5
N2—Zn2—S4	105.61 (15)	C25—C26—H26B	109.5
O13—Zn2—S4	99.79 (13)	H26A—C26—H26B	109.5
N2—Zn2—S3	109.24 (15)	C25—C26—H26C	109.5
O13—Zn2—S3	100.81 (13)	H26A—C26—H26C	109.5
S4—Zn2—S3	137.87 (6)	H26B—C26—H26C	109.5
Si1—S1—Zn1	85.98 (7)	C25—C27—H27A	109.5
Si2—S2—Zn1	89.35 (8)	C25—C27—H27B	109.5
Si3—S3—Zn2	93.88 (8)	H27A—C27—H27B	109.5
Si4—S4—Zn2	99.32 (8)	C25—C27—H27C	109.5
O3—Si1—O2	105.5 (2)	H27A—C27—H27C	109.5
O3—Si1—O1	113.5 (2)	H27B—C27—H27C	109.5
O2—Si1—O1	104.0 (2)	C25—C28—H28A	109.5
O3—Si1—S1	115.46 (17)	C25—C28—H28B	109.5
O2—Si1—S1	116.22 (19)	H28A—C28—H28B	109.5
O1—Si1—S1	101.86 (16)	C25—C28—H28C	109.5
O5—Si2—O6	104.6 (2)	H28A—C28—H28C	109.5
O5—Si2—O4	113.1 (2)	H28B—C28—H28C	109.5
O6—Si2—O4	106.3 (2)	O8—C29—C30	114.4 (8)
O5—Si2—S2	114.96 (19)	O8—C29—C31	106.0 (8)
O6—Si2—S2	115.06 (18)	C30—C29—C31	113.8 (9)
O4—Si2—S2	102.83 (17)	O8—C29—C32	105.5 (8)
O9—Si3—O8	104.5 (3)	C30—C29—C32	107.6 (9)
O9—Si3—O7	112.0 (2)	C31—C29—C32	109.2 (9)
O8—Si3—O7	105.8 (2)	C29—C30—H30A	109.5
O9—Si3—S3	105.60 (19)	C29—C30—H30B	109.5
O8—Si3—S3	112.98 (19)	H30A—C30—H30B	109.5
O7—Si3—S3	115.4 (2)	C29—C30—H30C	109.5
O12—Si4—O11	114.3 (2)	H30A—C30—H30C	109.5
O12—Si4—O10	104.5 (2)	H30B—C30—H30C	109.5
O11—Si4—O10	104.8 (2)	C29—C31—H31A	109.5
O12—Si4—S4	107.98 (19)	C29—C31—H31B	109.5
O11—Si4—S4	112.2 (2)	H31A—C31—H31B	109.5
O10—Si4—S4	112.91 (18)	C29—C31—H31C	109.5
C1—O1—Si1	130.4 (4)	H31A—C31—H31C	109.5
C1—O1—Zn1	133.6 (3)	H31B—C31—H31C	109.5
Si1—O1—Zn1	93.64 (18)	C29—C32—H32A	109.5
C5—O2—Si1	132.1 (4)	C29—C32—H32B	109.5
C9—O3—Si1	134.8 (4)	H32A—C32—H32B	109.5
C13—O4—Si2	130.6 (4)	C29—C32—H32C	109.5
C13—O4—Zn1	138.0 (4)	H32A—C32—H32C	109.5
Si2—O4—Zn1	91.27 (19)	H32B—C32—H32C	109.5
C17—O5—Si2	135.3 (4)	C35—C33—C36A	67.8 (13)
C21—O6—Si2	131.4 (4)	C35—C33—C34A	134.3 (13)
C25—O7—Si3	132.4 (4)	C36A—C33—C34A	119.3 (15)
C29—O8—Si3	133.7 (5)	C35—C33—O9	111.9 (8)
C33—O9—Si3	131.4 (5)	C36A—C33—O9	111.3 (9)
C37—O10—Si4	129.9 (4)	C34A—C33—O9	106.5 (11)
C41—O11—Si4	133.4 (4)	C35—C33—C34	111.8 (12)
C45—O12—Si4	135.0 (6)	C36A—C33—C34	133.8 (14)
C59—N1—C49	118.0 (5)	O9—C33—C34	110.8 (11)
C59—N1—Zn1	119.5 (4)	C36A—C33—C35A	110.0 (13)
C49—N1—Zn1	122.2 (4)	C34A—C33—C35A	108.0 (13)
C54—N2—C56	118.0 (6)	O9—C33—C35A	100.0 (8)
C54—N2—Zn2	120.7 (4)	C35—C33—C36	106.5 (10)
C56—N2—Zn2	121.2 (4)	C34A—C33—C36	80.7 (13)
O1—C1—C2	110.1 (5)	O9—C33—C36	111.3 (7)
O1—C1—C3	105.5 (5)	C34—C33—C36	104.2 (12)
C2—C1—C3	111.3 (7)	C33—C34—H34A	109.5
O1—C1—C4	109.2 (6)	C33—C34—H34B	109.5
C2—C1—C4	110.1 (6)	C33—C34—H34C	109.5
C3—C1—C4	110.5 (6)	C33—C35—H35A	109.5
C1—C2—H2A	109.5	C33—C35—H35B	109.5
C1—C2—H2B	109.5	C33—C35—H35C	109.5
H2A—C2—H2B	109.5	C33—C36—H36A	109.5
C1—C2—H2C	109.5	C33—C36—H36B	109.5
H2A—C2—H2C	109.5	C33—C36—H36C	109.5
H2B—C2—H2C	109.5	C33—C34A—H34D	109.5
C1—C3—H3A	109.5	C33—C34A—H34E	109.5
C1—C3—H3B	109.5	H34D—C34A—H34E	109.5
H3A—C3—H3B	109.5	C33—C34A—H34F	109.5
C1—C3—H3C	109.5	H34D—C34A—H34F	109.5
H3A—C3—H3C	109.5	H34E—C34A—H34F	109.5
H3B—C3—H3C	109.5	C33—C35A—H35D	109.5
C1—C4—H4A	109.5	C33—C35A—H35E	109.5
C1—C4—H4B	109.5	H35D—C35A—H35E	109.5
H4A—C4—H4B	109.5	C33—C35A—H35F	109.5
C1—C4—H4C	109.5	H35D—C35A—H35F	109.5
H4A—C4—H4C	109.5	H35E—C35A—H35F	109.5
H4B—C4—H4C	109.5	C33—C36A—H36D	109.5
O2—C5—C8	111.1 (5)	C33—C36A—H36E	109.5
O2—C5—C6	108.7 (6)	H36D—C36A—H36E	109.5
C8—C5—C6	110.9 (6)	C33—C36A—H36F	109.5
O2—C5—C7	105.5 (5)	H36D—C36A—H36F	109.5
C8—C5—C7	110.9 (7)	H36E—C36A—H36F	109.5
C6—C5—C7	109.6 (7)	O10—C37—C38	105.1 (5)
C5—C6—H6A	109.5	O10—C37—C39	110.6 (6)
C5—C6—H6B	109.5	C38—C37—C39	111.1 (6)
H6A—C6—H6B	109.5	O10—C37—C40	108.6 (5)
C5—C6—H6C	109.5	C38—C37—C40	109.2 (7)
H6A—C6—H6C	109.5	C39—C37—C40	111.8 (7)
H6B—C6—H6C	109.5	C37—C38—H38A	109.5
C5—C7—H7A	109.5	C37—C38—H38B	109.5
C5—C7—H7B	109.5	H38A—C38—H38B	109.5
H7A—C7—H7B	109.5	C37—C38—H38C	109.5
C5—C7—H7C	109.5	H38A—C38—H38C	109.5
H7A—C7—H7C	109.5	H38B—C38—H38C	109.5
H7B—C7—H7C	109.5	C37—C39—H39A	109.5
C5—C8—H8A	109.5	C37—C39—H39B	109.5
C5—C8—H8B	109.5	H39A—C39—H39B	109.5
H8A—C8—H8B	109.5	C37—C39—H39C	109.5
C5—C8—H8C	109.5	H39A—C39—H39C	109.5
H8A—C8—H8C	109.5	H39B—C39—H39C	109.5
H8B—C8—H8C	109.5	C37—C40—H40A	109.5
O3—C9—C12	110.7 (5)	C37—C40—H40B	109.5
O3—C9—C10	104.9 (5)	H40A—C40—H40B	109.5
C12—C9—C10	111.4 (7)	C37—C40—H40C	109.5
O3—C9—C11	108.7 (6)	H40A—C40—H40C	109.5
C12—C9—C11	110.0 (6)	H40B—C40—H40C	109.5
C10—C9—C11	110.8 (6)	O11—C41—C42	109.1 (5)
C9—C10—H10A	109.5	O11—C41—C44	110.7 (5)
C9—C10—H10B	109.5	C42—C41—C44	110.6 (7)
H10A—C10—H10B	109.5	O11—C41—C43	104.6 (6)
C9—C10—H10C	109.5	C42—C41—C43	111.4 (7)
H10A—C10—H10C	109.5	C44—C41—C43	110.4 (6)
H10B—C10—H10C	109.5	C41—C42—H42A	109.5
C9—C11—H11A	109.5	C41—C42—H42B	109.5
C9—C11—H11B	109.5	H42A—C42—H42B	109.5
H11A—C11—H11B	109.5	C41—C42—H42C	109.5
C9—C11—H11C	109.5	H42A—C42—H42C	109.5
H11A—C11—H11C	109.5	H42B—C42—H42C	109.5
H11B—C11—H11C	109.5	C41—C43—H43A	109.5
C9—C12—H12A	109.5	C41—C43—H43B	109.5
C9—C12—H12B	109.5	H43A—C43—H43B	109.5
H12A—C12—H12B	109.5	C41—C43—H43C	109.5
C9—C12—H12C	109.5	H43A—C43—H43C	109.5
H12A—C12—H12C	109.5	H43B—C43—H43C	109.5
H12B—C12—H12C	109.5	C41—C44—H44A	109.5
O4—C13—C14	106.1 (5)	C41—C44—H44B	109.5
O4—C13—C16	110.1 (5)	H44A—C44—H44B	109.5
C14—C13—C16	110.4 (6)	C41—C44—H44C	109.5
O4—C13—C15	107.6 (5)	H44A—C44—H44C	109.5
C14—C13—C15	110.9 (6)	H44B—C44—H44C	109.5
C16—C13—C15	111.5 (6)	O12—C45—C48	113.2 (8)
C13—C14—H14A	109.5	O12—C45—C46	106.5 (8)
C13—C14—H14B	109.5	C48—C45—C46	110.7 (10)
H14A—C14—H14B	109.5	O12—C45—C47	106.0 (8)
C13—C14—H14C	109.5	C48—C45—C47	113.8 (9)
H14A—C14—H14C	109.5	C46—C45—C47	106.1 (9)
H14B—C14—H14C	109.5	C45—C46—H46A	109.5
C13—C15—H15A	109.5	C45—C46—H46B	109.5
C13—C15—H15B	109.5	H46A—C46—H46B	109.5
H15A—C15—H15B	109.5	C45—C46—H46C	109.5
C13—C15—H15C	109.5	H46A—C46—H46C	109.5
H15A—C15—H15C	109.5	H46B—C46—H46C	109.5
H15B—C15—H15C	109.5	C45—C47—H47A	109.5
C13—C16—H16A	109.5	C45—C47—H47B	109.5
C13—C16—H16B	109.5	H47A—C47—H47B	109.5
H16A—C16—H16B	109.5	C45—C47—H47C	109.5
C13—C16—H16C	109.5	H47A—C47—H47C	109.5
H16A—C16—H16C	109.5	H47B—C47—H47C	109.5
H16B—C16—H16C	109.5	C45—C48—H48A	109.5
O5—C17—C18	110.9 (6)	C45—C48—H48B	109.5
O5—C17—C19	109.3 (6)	H48A—C48—H48B	109.5
C18—C17—C19	112.4 (7)	C45—C48—H48C	109.5
O5—C17—C20	104.4 (6)	H48A—C48—H48C	109.5
C18—C17—C20	109.7 (8)	H48B—C48—H48C	109.5
C19—C17—C20	109.9 (7)	N1—C49—C50	122.9 (6)
C17—C18—H18A	109.5	N1—C49—H49	118.5
C17—C18—H18B	109.5	C50—C49—H49	118.5
H18A—C18—H18B	109.5	C49—C50—C51	119.4 (5)
C17—C18—H18C	109.5	C49—C50—H50	120.3
H18A—C18—H18C	109.5	C51—C50—H50	120.3
H18B—C18—H18C	109.5	C58—C51—C50	117.2 (5)
C17—C19—H19A	109.5	C58—C51—C52	120.9 (6)
C17—C19—H19B	109.5	C50—C51—C52	121.7 (5)
H19A—C19—H19B	109.5	C53—C52—C57	117.5 (6)
C17—C19—H19C	109.5	C53—C52—C51	121.6 (6)
H19A—C19—H19C	109.5	C57—C52—C51	120.7 (6)
H19B—C19—H19C	109.5	C54—C53—C52	119.8 (6)
C17—C20—H20A	109.5	C54—C53—H53	120.1
C17—C20—H20B	109.5	C52—C53—H53	120.1
H20A—C20—H20B	109.5	N2—C54—C53	122.9 (6)
C17—C20—H20C	109.5	N2—C54—H54	118.6
H20A—C20—H20C	109.5	C53—C54—H54	118.6
H20B—C20—H20C	109.5	N2—C56—C57	122.9 (6)
O6—C21—C22	110.7 (5)	N2—C56—H56	118.6
O6—C21—C23	110.3 (6)	C57—C56—H56	118.6
C22—C21—C23	111.0 (6)	C56—C57—C52	118.9 (6)
O6—C21—C24	105.2 (5)	C56—C57—H57	120.6
C22—C21—C24	110.0 (6)	C52—C57—H57	120.6
C23—C21—C24	109.5 (6)	C59—C58—C51	119.9 (6)
C21—C22—H22A	109.5	C59—C58—H58	120
C21—C22—H22B	109.5	C51—C58—H58	120
H22A—C22—H22B	109.5	N1—C59—C58	122.5 (5)
C21—C22—H22C	109.5	N1—C59—H59	118.7
H22A—C22—H22C	109.5	C58—C59—H59	118.7
H22B—C22—H22C	109.5	O13—C60—H60A	109.5
C21—C23—H23A	109.5	O13—C60—H60B	109.5
C21—C23—H23B	109.5	H60A—C60—H60B	109.5
H23A—C23—H23B	109.5	O13—C60—H60C	109.5
C21—C23—H23C	109.5	H60A—C60—H60C	109.5
H23A—C23—H23C	109.5	H60B—C60—H60C	109.5
H23B—C23—H23C	109.5	C60—O13—Zn2	124.5 (4)
C21—C24—H24A	109.5	C60—O13—H13A	111 (5)
C21—C24—H24B	109.5	Zn2—O13—H13A	117 (4)
			
N1—Zn1—S1—Si1	84.07 (16)	O4—Zn1—N1—C59	−102.5 (5)
S2—Zn1—S1—Si1	−109.63 (11)	S2—Zn1—N1—C49	−2.2 (5)
O1—Zn1—S1—Si1	−4.64 (12)	S1—Zn1—N1—C49	168.3 (4)
O4—Zn1—S1—Si1	176.98 (12)	O1—Zn1—N1—C49	−113.6 (5)
N1—Zn1—S2—Si2	92.88 (17)	O4—Zn1—N1—C49	71.6 (5)
S1—Zn1—S2—Si2	−73.10 (12)	O13—Zn2—N2—C54	−87.8 (5)
O1—Zn1—S2—Si2	−166.61 (12)	S4—Zn2—N2—C54	13.4 (5)
O4—Zn1—S2—Si2	8.02 (11)	S3—Zn2—N2—C54	169.4 (5)
N2—Zn2—S3—Si3	−73.62 (17)	O13—Zn2—N2—C56	96.7 (5)
O13—Zn2—S3—Si3	−171.46 (13)	S4—Zn2—N2—C56	−162.1 (4)
S4—Zn2—S3—Si3	70.56 (12)	S3—Zn2—N2—C56	−6.1 (5)
N2—Zn2—S4—Si4	−141.77 (16)	Si1—O1—C1—C2	−42.1 (8)
O13—Zn2—S4—Si4	−45.08 (15)	Zn1—O1—C1—C2	160.4 (5)
S3—Zn2—S4—Si4	73.25 (12)	Si1—O1—C1—C3	−162.2 (5)
Zn1—S1—Si1—O3	130.0 (2)	Zn1—O1—C1—C3	40.3 (8)
Zn1—S1—Si1—O2	−105.65 (18)	Si1—O1—C1—C4	79.0 (6)
Zn1—S1—Si1—O1	6.58 (17)	Zn1—O1—C1—C4	−78.5 (7)
Zn1—S2—Si2—O5	111.0 (2)	Si1—O2—C5—C8	40.1 (9)
Zn1—S2—Si2—O6	−127.46 (19)	Si1—O2—C5—C6	−82.2 (7)
Zn1—S2—Si2—O4	−12.39 (17)	Si1—O2—C5—C7	160.4 (6)
Zn2—S3—Si3—O9	10.0 (2)	Si1—O3—C9—C12	−44.4 (9)
Zn2—S3—Si3—O8	123.7 (2)	Si1—O3—C9—C10	−164.8 (6)
Zn2—S3—Si3—O7	−114.32 (19)	Si1—O3—C9—C11	76.6 (7)
Zn2—S4—Si4—O12	−49.28 (19)	Si2—O4—C13—C14	−158.0 (5)
Zn2—S4—Si4—O11	−176.06 (18)	Zn1—O4—C13—C14	28.1 (8)
Zn2—S4—Si4—O10	65.8 (2)	Si2—O4—C13—C16	−38.6 (7)
O3—Si1—O1—C1	65.0 (6)	Zn1—O4—C13—C16	147.6 (5)
O2—Si1—O1—C1	−49.0 (6)	Si2—O4—C13—C15	83.2 (6)
S1—Si1—O1—C1	−170.2 (5)	Zn1—O4—C13—C15	−90.7 (6)
O3—Si1—O1—Zn1	−131.1 (2)	Si2—O5—C17—C18	−43.3 (9)
O2—Si1—O1—Zn1	114.8 (2)	Si2—O5—C17—C19	81.1 (8)
S1—Si1—O1—Zn1	−6.34 (16)	Si2—O5—C17—C20	−161.4 (6)
N1—Zn1—O1—C1	61.1 (5)	Si2—O6—C21—C22	40.5 (8)
S2—Zn1—O1—C1	−52.5 (5)	Si2—O6—C21—C23	−82.7 (7)
S1—Zn1—O1—C1	168.8 (5)	Si2—O6—C21—C24	159.3 (5)
N1—Zn1—O1—Si1	−101.9 (2)	Si3—O7—C25—C28	33.0 (8)
S2—Zn1—O1—Si1	144.49 (14)	Si3—O7—C25—C27	−89.5 (6)
S1—Zn1—O1—Si1	5.79 (15)	Si3—O7—C25—C26	152.3 (5)
O3—Si1—O2—C5	45.9 (6)	Si3—O8—C29—C30	−8.7 (14)
O1—Si1—O2—C5	165.6 (6)	Si3—O8—C29—C31	−134.9 (7)
S1—Si1—O2—C5	−83.4 (6)	Si3—O8—C29—C32	109.3 (8)
O2—Si1—O3—C9	−163.3 (6)	Si3—O9—C33—C35	103.0 (10)
O1—Si1—O3—C9	83.5 (6)	Si3—O9—C33—C36A	29.3 (15)
S1—Si1—O3—C9	−33.6 (6)	Si3—O9—C33—C34A	−102.3 (13)
O5—Si2—O4—C13	70.5 (5)	Si3—O9—C33—C34	−131.5 (11)
O6—Si2—O4—C13	−43.7 (5)	Si3—O9—C33—C35A	145.4 (9)
S2—Si2—O4—C13	−164.9 (5)	Si3—O9—C33—C36	−16.0 (11)
O5—Si2—O4—Zn1	−113.6 (2)	Si4—O10—C37—C38	−163.9 (5)
O6—Si2—O4—Zn1	132.24 (19)	Si4—O10—C37—C39	−43.8 (8)
S2—Si2—O4—Zn1	10.98 (15)	Si4—O10—C37—C40	79.3 (8)
N1—Zn1—O4—C13	53.2 (5)	Si4—O11—C41—C42	−84.6 (7)
S2—Zn1—O4—C13	165.1 (5)	Si4—O11—C41—C44	37.3 (9)
S1—Zn1—O4—C13	−54.9 (5)	Si4—O11—C41—C43	156.2 (6)
N1—Zn1—O4—Si2	−122.2 (2)	Si4—O12—C45—C48	17.0 (14)
S2—Zn1—O4—Si2	−10.24 (14)	Si4—O12—C45—C46	−104.8 (9)
S1—Zn1—O4—Si2	129.79 (16)	Si4—O12—C45—C47	142.5 (7)
O6—Si2—O5—C17	−170.3 (6)	C59—N1—C49—C50	2.3 (9)
O4—Si2—O5—C17	74.6 (7)	Zn1—N1—C49—C50	−171.9 (5)
S2—Si2—O5—C17	−43.1 (7)	N1—C49—C50—C51	0.3 (10)
O5—Si2—O6—C21	38.7 (6)	C49—C50—C51—C58	−3.2 (9)
O4—Si2—O6—C21	158.6 (5)	C49—C50—C51—C52	172.0 (6)
S2—Si2—O6—C21	−88.4 (5)	C58—C51—C52—C53	146.2 (7)
O9—Si3—O7—C25	−80.1 (6)	C50—C51—C52—C53	−28.8 (10)
O8—Si3—O7—C25	166.6 (5)	C58—C51—C52—C57	−30.0 (9)
S3—Si3—O7—C25	40.9 (6)	C50—C51—C52—C57	155.1 (6)
O9—Si3—O8—C29	−177.0 (8)	C57—C52—C53—C54	1.8 (10)
O7—Si3—O8—C29	−58.6 (8)	C51—C52—C53—C54	−174.5 (6)
S3—Si3—O8—C29	68.7 (8)	C56—N2—C54—C53	−2.4 (10)
O8—Si3—O9—C33	59.0 (6)	Zn2—N2—C54—C53	−178.0 (5)
O7—Si3—O9—C33	−55.1 (6)	C52—C53—C54—N2	0.3 (10)
S3—Si3—O9—C33	178.5 (5)	C54—N2—C56—C57	2.3 (9)
O12—Si4—O10—C37	−159.1 (5)	Zn2—N2—C56—C57	178.0 (5)
O11—Si4—O10—C37	−38.6 (6)	N2—C56—C57—C52	−0.2 (10)
S4—Si4—O10—C37	83.8 (5)	C53—C52—C57—C56	−1.8 (10)
O12—Si4—O11—C41	−80.9 (6)	C51—C52—C57—C56	174.5 (6)
O10—Si4—O11—C41	165.3 (6)	C50—C51—C58—C59	3.6 (9)
S4—Si4—O11—C41	42.4 (6)	C52—C51—C58—C59	−171.6 (6)
O11—Si4—O12—C45	−62.1 (9)	C49—N1—C59—C58	−1.9 (9)
O10—Si4—O12—C45	51.9 (9)	Zn1—N1—C59—C58	172.5 (5)
S4—Si4—O12—C45	172.3 (8)	C51—C58—C59—N1	−1.1 (10)
S2—Zn1—N1—C59	−176.4 (4)	N2—Zn2—O13—C60	−92.0 (5)
S1—Zn1—N1—C59	−5.9 (5)	S4—Zn2—O13—C60	161.5 (5)
O1—Zn1—N1—C59	72.3 (5)	S3—Zn2—O13—C60	18.4 (5)

### Hydrogen-bond geometry (Å, °)

**Table:**

*D*—H···*A*	*D*—H	H···*A*	*D*···*A*	*D*—H···*A*
O13—H13A···O10	0.99 (1)	1.86 (4)	2.771 (6)	151 (6)
